# Supplementary material for: The role of results in deciding to publish: A direct comparison across authors, reviewers, and editors based on an online survey
Source: PLoS One. 2023 Oct 3;18(10):e0292279. doi: 10.1371/journal.pone.0292279 (PMC10547160; doi:10.1371/journal.pone.0292279)
Supplement: S1 Table — (DOCX) [file pone.0292279.s001.docx]

| **Scientific role** | **Scenario** | **Questions about the significant result** | **Questions about the non-significant result** |
| --- | --- | --- | --- |
| **Editors** | “Suppose two papers have been *submitted* to your journal. Both of the papers fit very well in your journal in terms of content, and both of the papers have a rigorous introduction, method, results, and discussion section. The main result of one of the papers is statistically significant at an alpha level of 5% (*p* = .02) and the main result of the other paper is statistically non-significant (*p* = .26).” | “How likely do you think it is for you to *accept* the paper with the statistically significant main result for publication in your journal on a scale ranging from 0 (0% likely to *accept* the paper) to 100 (100% likely to *accept* the paper)?” | “How likely do you think it is for you to *accept* the paper with the statistically non-significant main result for publication in your journal on a scale ranging from 0 (0% likely to *accept* the paper) to 100 (100% likely to *accept* the paper)?” |
| **Reviewers** | “Suppose you are asked to *review* two papers that have been submitted to a journal in your field. Both of the papers fit very well in the journal in terms of content, and both of the papers have a rigorous introduction, method, results, and discussion section. The main result of one of the papers is statistically significant at an alpha level of 5% (*p* = .02) and the main result of the other paper is statistically non-significant (*p* = .26).” | “How likely do you think it is for you to *recommend* publication of the paper with the statistically significant main result on a scale ranging from 0 (0% likely to *recommend* publication of the paper) to 100 (100% likely to *recommend* publication of the paper)?” | “How likely do you think it is for you to *recommend* publication of the paper with the statistically non-significant main result on a scale ranging from 0 (0% likely to *recommend* publication of the paper) to 100 (100% likely to *recommend* publication of the paper)?” |
| **Authors** | “Suppose you have been *working* on two different studies. Both of the studies have a high methodological quality. The main result of one of the studies is statistically significant at an alpha level of 5% (*p* = .02) and the main result of the other study is statistically non-significant (*p* = .26).” | “How likely do you think it is for you to *write up* the study with the statistically significant main result on a scale ranging from 0 (0% likely to *write up* the study) to 100 (100% likely to *write up* the study)?” | “How likely do you think it is for you to *write up* the study with the statistically non-significant main result on a scale ranging from 0 (0% likely to *write up* the study) to 100 (100% likely to *write up* the study)?” |
|  |  | “How likely do you think it is for you to *submit* the paper with the statistically significant main result for publication on a scale ranging from 0 (0% likely to *submit* the paper) to 100 (100% likely to *submit* the paper)?” | “How likely do you think it is for you to *submit* the paper with the statistically non-significant main result for publication on a scale ranging from 0 (0% likely to *submit* the paper) to 100 (100% likely to *submit* the paper)?” |

*Note*: The important differences between the scenarios and the questions across the different scientific roles are underlined and in italic. This is done for the reader only (meaning that the participants did not see this); in order to measure the likelihood of endorsing publication, the editors received two questions; the reviewers received two questions; the authors received four questions (for our analyses, we computed the average score for significant and non-significant results based on the “write up” and “submit” questions). The order of the questions about the significant and the non-significant result varied: approximately half of the respondents was presented with the significant result first, and the other half was presented with the question about the non-significant result first.
