# Supplementary figures and images for: The role of results in deciding to publish: A direct comparison across authors, reviewers, and editors based on an online survey
Source: PLoS One. 2023 Oct 3;18(10):e0292279. doi: 10.1371/journal.pone.0292279 (PMC10547160; doi:10.1371/journal.pone.0292279)

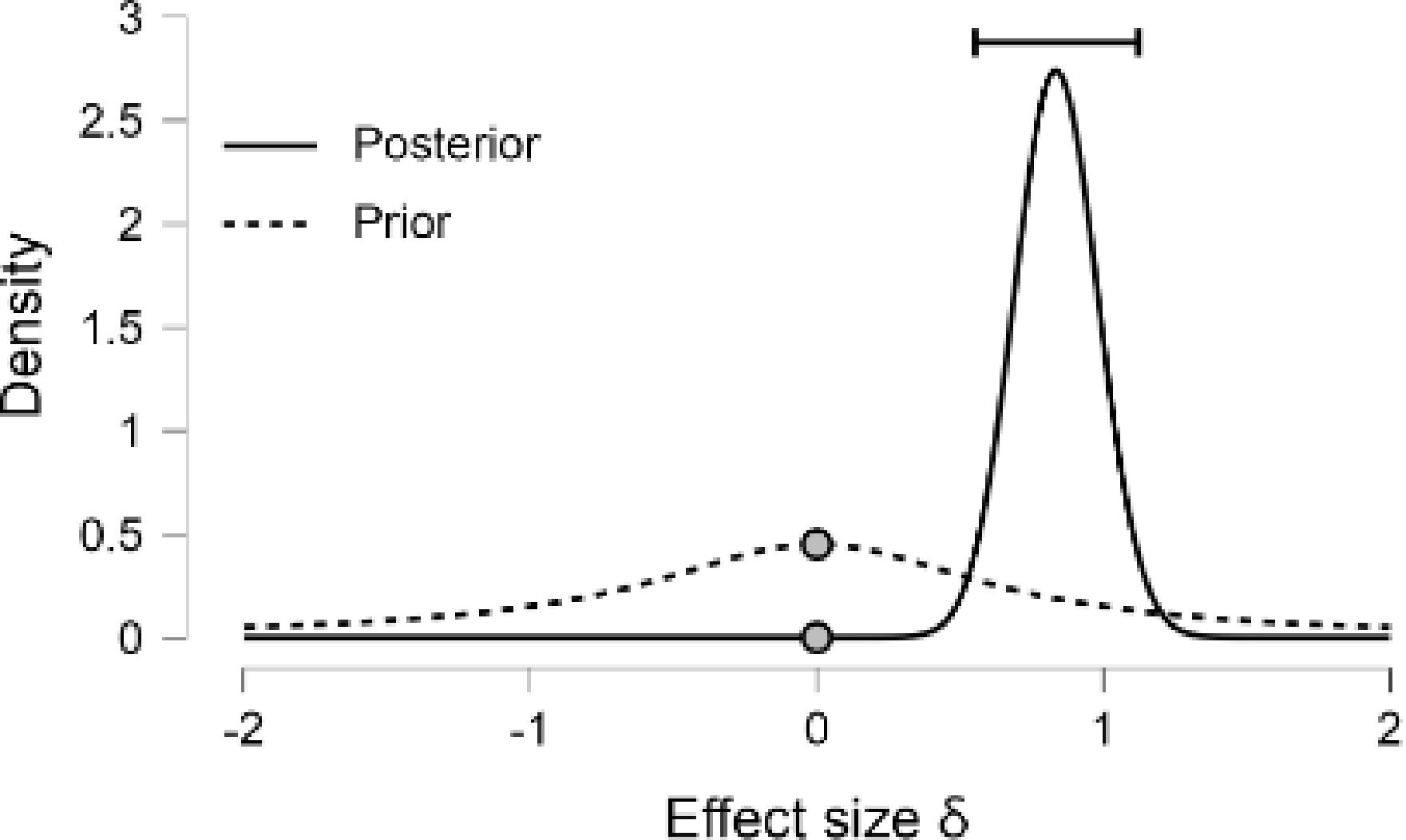

Supplement: S1 Fig — (TIF) [file pone.0292279.s002.tif]

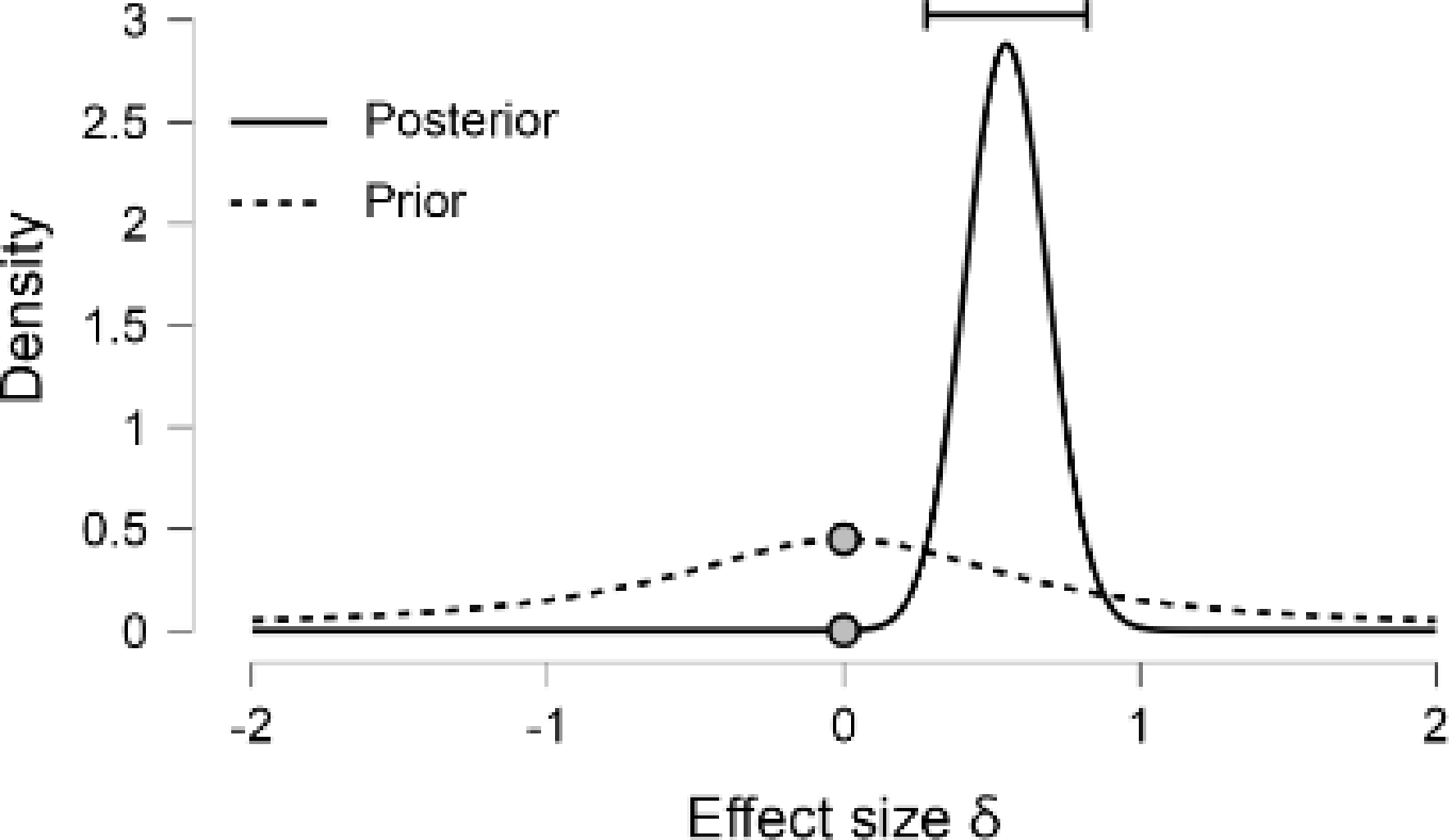

Supplement: S2 Fig — (TIF) [file pone.0292279.s003.tif]

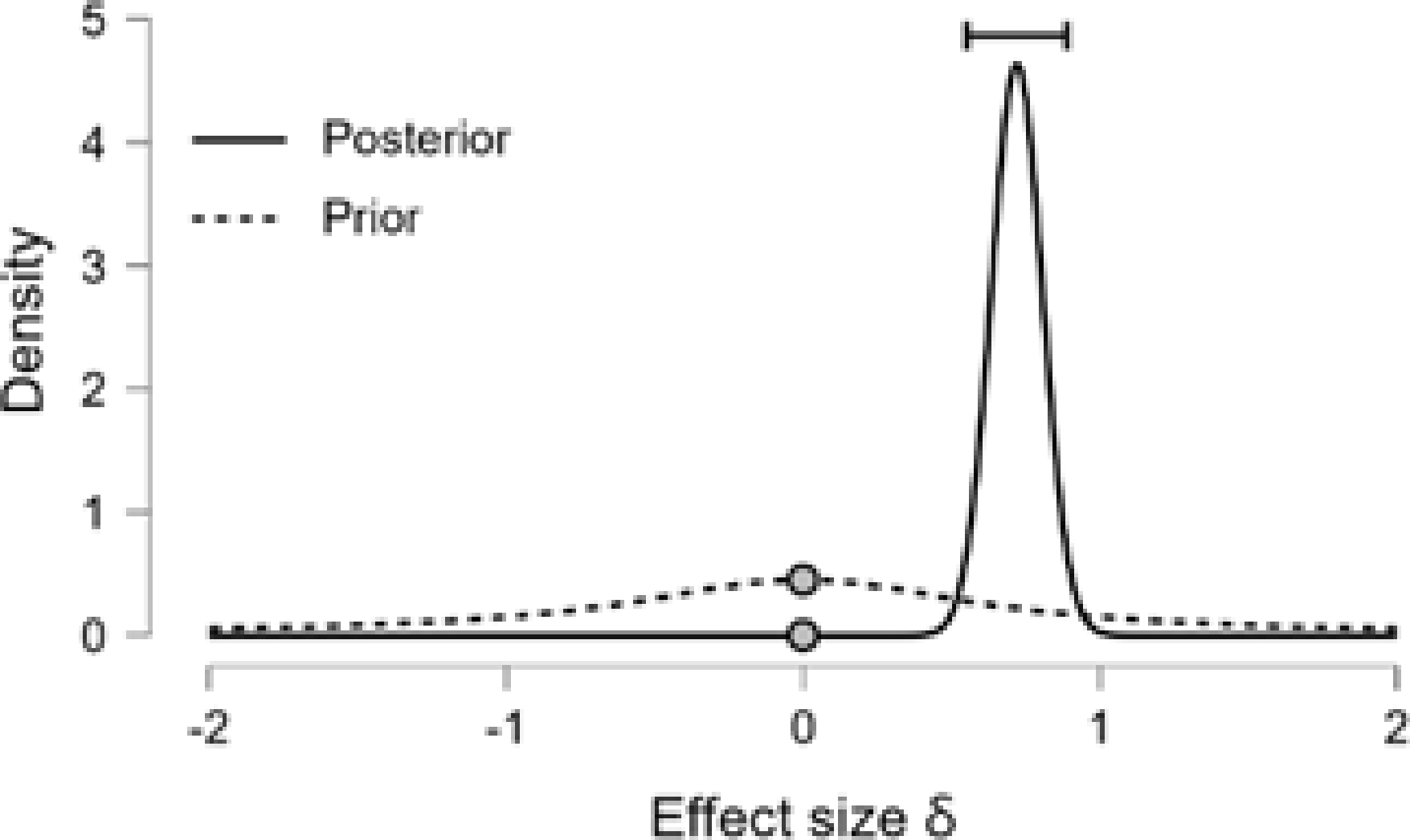

Supplement: S3 Fig — (TIF) [file pone.0292279.s004.tif]

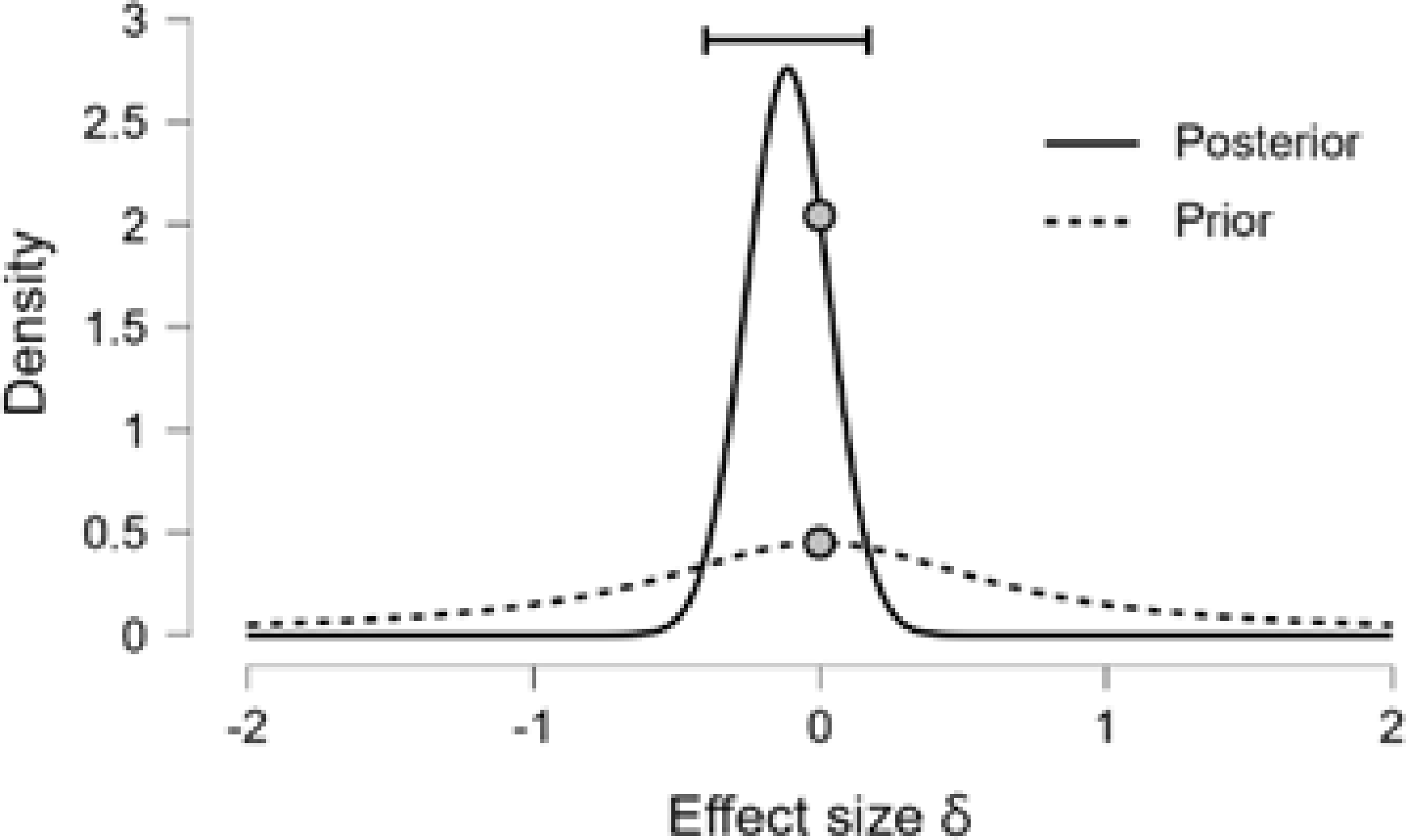

Supplement: S4 Fig — (TIF) [file pone.0292279.s005.tif]

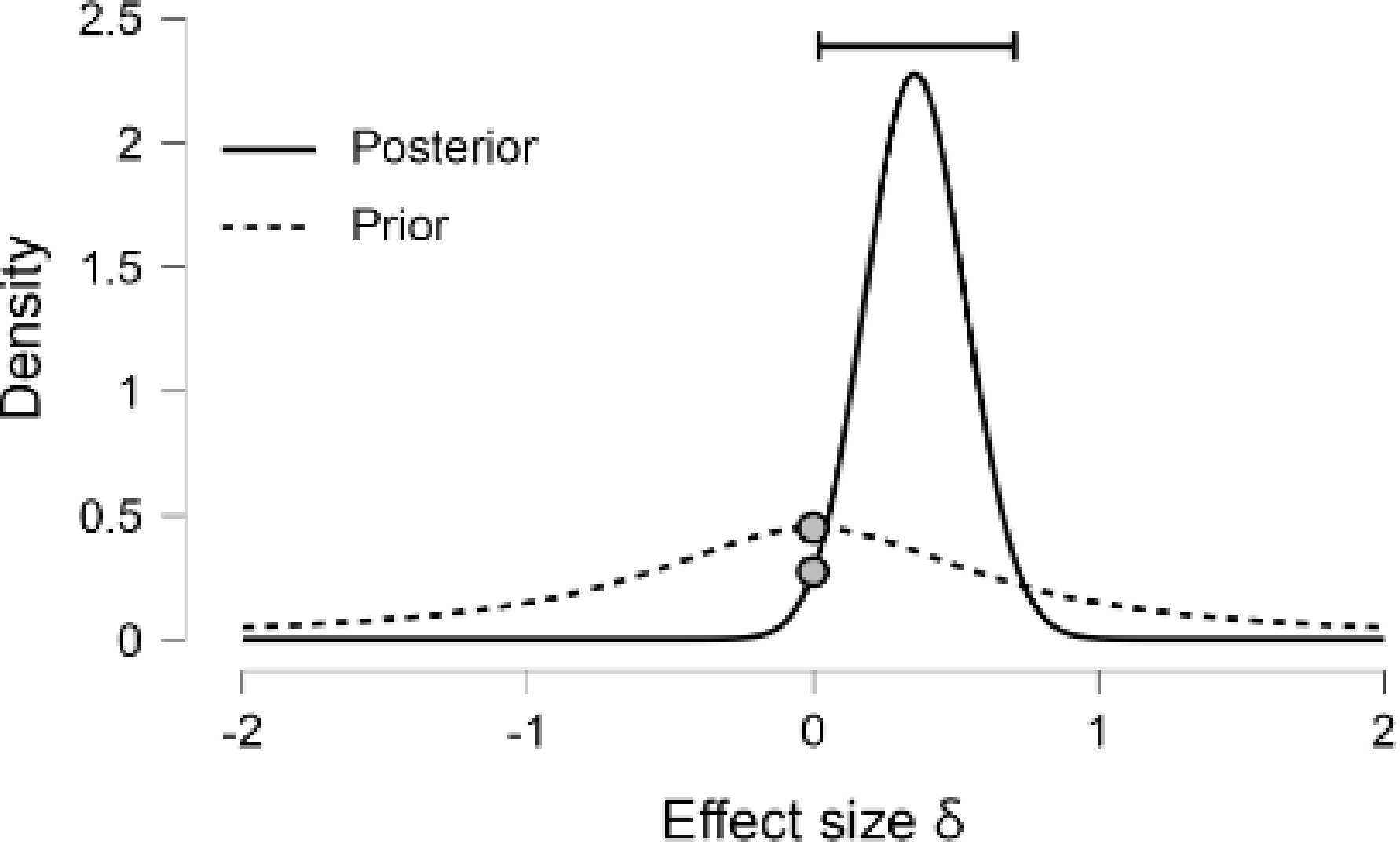

Supplement: S5 Fig — (TIF) [file pone.0292279.s006.tif]

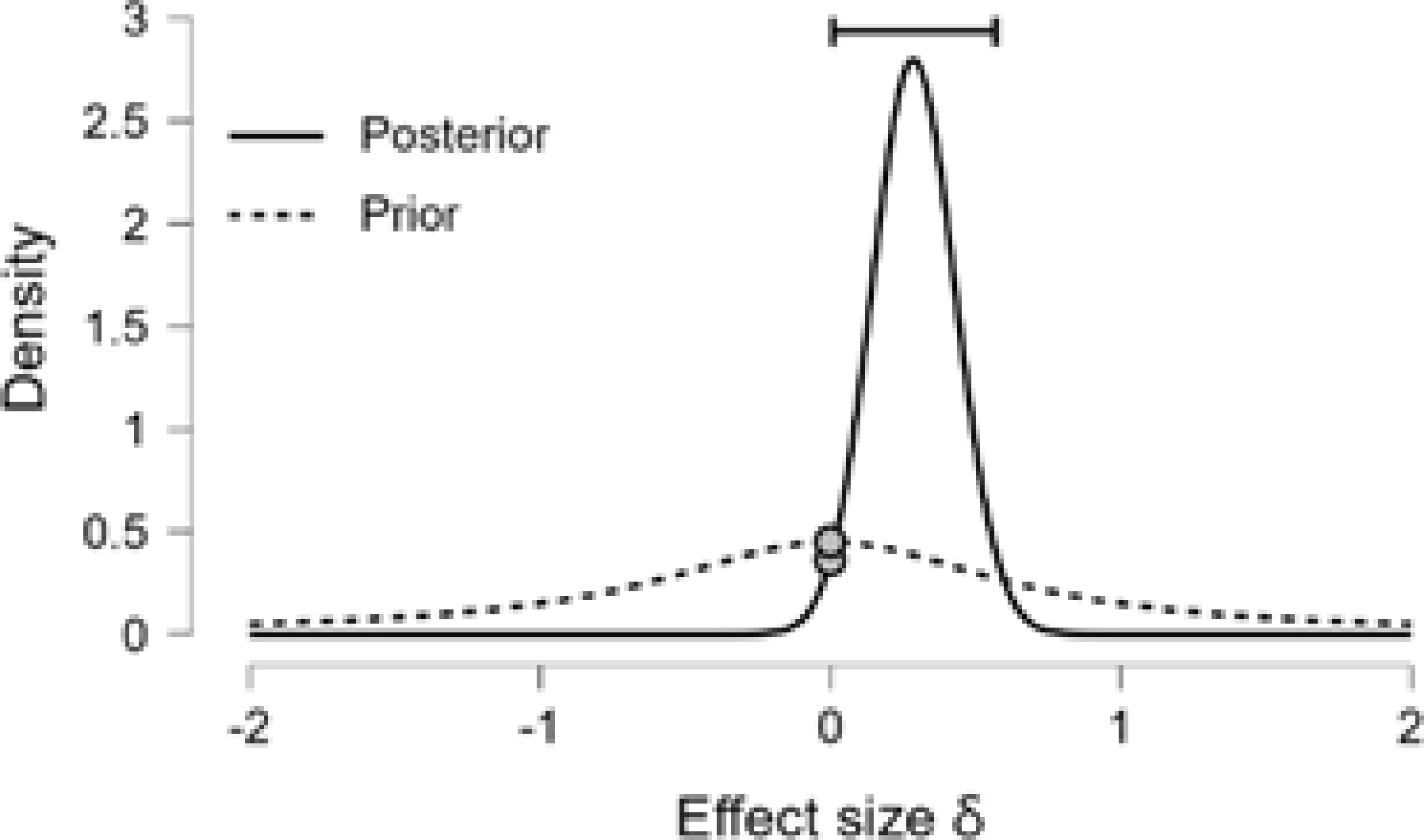

Supplement: S6 Fig — (TIF) [file pone.0292279.s007.tif]
