# Supplementary material for: The role of results in deciding to publish: A direct comparison across authors, reviewers, and editors based on an online survey
Source: PLoS One. 2023 Oct 3;18(10):e0292279. doi: 10.1371/journal.pone.0292279 (PMC10547160; doi:10.1371/journal.pone.0292279)
Supplement: Attachment — Submitted filename: Response to Reviewers.docx [file pone.0292279.s009.docx]

**Editor**

Thank you for submitting your manuscript to PLOS ONE. After careful consideration, we feel that it has merit but does not fully meet PLOS ONE’s publication criteria as it currently stands. Therefore, we invite you to submit a revised version of the manuscript that addresses the points raised during the review process.

First of all, I would like to thank the 2 reviewers who assessed this new version of the manuscript. The third reviewer was unavailable but I could check your answers to this reviewer and think that you answered appropriately to this reviewer. One of the 2 remaining reviewers has remaining questions/requests for clarifications that need to be addressed before I can recommend acceptance.

In addition I have the following requests:

- Please mention in the title the study design ;

- Please mention in the abstract the response rate to the survey as an additional limitation ;

- To enhance reporting, please follow an adequate reporting guideline both for the manuscript and the abstract ;

**E-1: We would like to thank you and the reviewers for reading our manuscript and for the comments.**

**We have revised the title. The new title is: “The role of results in deciding to publish****: A direct comparison across authors, reviewers, and editors based on an online survey”.**

**In the new abstract, at the end we mention the response rate to the survey as an additional limitation: “****Another limitation was the low response rate to the survey.”**

**To enhance reporting, we made some revisions in our reporting in the abstract and added subheadings to the abstract and manuscript.**

**Reviewer #2**

The article "Publication Bias in Scientific Literature: Implications and Strategies" has improved substantively compared to the first version. However, I still have some issues that should be addressed before publication:

**R2-0: Thank you for reading and commenting our manuscript again.**

Major remarks:

1. The sample composition l. 138 could be reported more clearly, describing the editor and the dependent author/reviewer sample along with the removed duplicates (at best in a table). Please also specify the number of sample persons separately for authors and reviewers (l. 141). The response rate is reported to be 10.3% for both conditions, assuming a half split (607,5) the response rates would be 9,9 or 10,6% (for the authors' sample, there have to be at least 629 sample persons to arrive at a response rate of 10.3%. Please specify the sample sizes and correct the response rate if necessary.

**R2-1: We have collected our data according to a stepwise procedure:** **1. We invited 1196 editors, of which 171 responded to the survey. 2. We invited 1215 researchers for the role of reviewer or author, of which 125 responded. 3. Qualtrics randomly assigned each of the respondents in step 2 to the roles of Author (65) and Reviewer (60). We added this information in the following underlined way to the method section (subsection “Participants”), and hopefully it is more clear now:**

**“In our analyses, we only included the answers of the respondents who answered the question about the significant result and the question about the non-significant result (see below in the subsection “Materials and procedure” for detailed information about the questions included in our surveys).** **We collected our samples in a stepwise way: 1) We invited 1196 editors, of which 171 responded to the survey. 2) We invited 1215 researchers for the role of reviewer or author, of which 125 responded. 3) Qualtrics randomly assigned each of the respondents in step 2 to the roles of Author (65) and Reviewer (60). The eventual sample sizes for authors, reviewers, and editors were 65, 60, and 171 respectively. The final response rate was 10.3% for the authors/reviewers and 14.3% for the editors.”**

2. Why does Table 4 only contain descriptive results, Fig. 2 in turn, is only mentioned in one sentence and seems to be completely unrelated to the research question

**R2-2: We have now added 95% confidence intervals for the average differences in Table 3. Additionally, we have added inferential information (BF_10_s and 95% credible intervals) when describing the results in Table 3. In order to see how we have performed these analyses (in JASP), see file “2023-08-11 Transparency Document.docx”. In the manuscript (at the end of section “Results”), we have added the following (underlined parts):**

**“For each category of the qualitative question, we computed the average DS in the likelihood of endorsing publication between the significant and the non-significant results (see Table 3 column 3).** **We performed exploratory analyses in which we conducted Bayesian independent samples *t* tests in JASP in order to test the difference in the average DS between category 1 (*p*-value is decisive) versus category 2 (*p*-value is important/relevant but not decisive), and between category 2 versus category 3 (*p*-value is not important/relevant). The null hypotheses for the two *t* tests stated that there is no difference in the average DS between the two categories, and the alternative hypotheses stated that there is a difference in the average DS between the two categories. For each test we conducted, we calculated Bayes factors that quantify the relative evidence for the alternative hypothesis over the null hypothesis (BF_10_) provided by the data, and 95% credible intervals based on posterior distributions of the standardized effect size parameters. The results showed that the higher the importance of the *p*-value indicated in the answer to the qualitative question, the larger the average DS in the likelihood of endorsing publication between the significant and the non-significant results (category 1 versus category 2: BF_10_ = 5.09*10^8^, 95% credible interval = 1.05, 1.97; category 2 versus category 3: BF_10_ = 4.02*10^5^, 95% credible interval = 0.54, 1.16). This means that the answers to the qualitative question were in line with the answers to the quantitative questions about the significant and non-significant results. Furthermore, it appeared that the average DS in the categories 4 (i.e., other/unclear [rest category]) and 0 (i.e., no answer provided to the qualitative question) was quite large too.”**

**Regarding Fig. 2, we think that it is important to include it in our manuscript, because in the preregistration we had mentioned that we would explore to what extent the difference in likelihood of accepting a significant versus a non-significant result was related to familiarity with significance testing. Since the scores on the familiarity with significance testing question appeared to have a ceiling effect, we decided to drop that analysis, and we would like to clarify to the reader why we decided to drop that analysis by showing that figure. However, we do understand that the text in which we mention Fig. 2 is too much separated from the preregistration. Therefore, we replaced the following part from our Results section:**

**“Finally, the scores on the question about respondents’ familiarity with significance testing are presented in Fig 2. It can be seen that there was not much variation in the answers to this question.”**

**by:**

**“As mentioned at the end of the method section, we were planning in our preregistration to explore to what extent the difference in likelihood of accepting a significant versus a non-significant result was related to familiarity with significance testing. We decided to drop this analysis because, as can be observed in Fig. 2, the scores on the familiarity with significance testing question appeared to have a ceiling effect.”**

3. I am still not entirely convinced by the use of Bayesian t-tests; what are the major advantages of using this methodology (does it outperform the frequentist t-test in respect of statistical power)? Please elaborate why in l, 207 this specific scale parameter was chosen.

**R2-3: Thank you for these comments. Our main argument for using Bayesian *t* tests (rather than frequentist *t* tests) is not because the Bayesian *t* test outperforms the frequentist *t* test in respect of statistical power. We decided to use Bayesian *t* tests because they allow for quantification of evidence in favour of the null hypothesis (relative to a composite alternative hypothesis), such as mentioned in subsection “Analyses”.**

**We used a Cauchy distribution centred on zero with scale parameter 1/√2, because this is the default prior which is often used in Bayesian null hypothesis testing, and thus makes results comparable across different studies. We agree that this information was missing from the manuscript, and thus we added it to subsection “Analyses” (see the underlined parts):**

**“The prior distribution for the standardized effect size under the alternative hypothesis was a Cauchy distribution centred on zero with scale parameter 1/√2,** **which is the default prior that is often used in Bayesian null hypothesis testing, and thus makes results comparable across different studies”.**

Minor remarks:

l. 161 replace “fabricated scenario” by “hypothetical scenario”

**R2-4: Thank you for this suggestion. We agree that “hypothetical scenario” sounds better, so we replaced “fabricated” by “hypothetical” in the manuscript.**

l 186 replace “difference score” by “risk difference” (the effect sizes name)

**R2-5: When writing the manuscript, we struggled with choosing a compatible term for this score. Although we understand that “difference score” might sound a bit vague, we think that “risk difference” may sound a bit negative. Therefore, we decided to make the following adjustment (see underlined parts) to the first paragraph in the subsection “Analyses”:**

**“... we first computed a difference score (in percentage points) in the likelihood of endorsing publication between the significant and the non-significant results for all respondents (from here on DS).”**

l, 207 why is exactly this scale parameter chosen?

**R2-6: Please see R2-3.**

l. 248 refer to the difference as percentage points

**R2-7: We agree with this comment. Please see R2-5.**

l. 279 and other Tables: Format of tables should be improved (esp. avoiding too many lines)

**R2-8: We have tried to create our tables according to the PLOS ONE formatting requirements as much as possible.**

l. 279 What specific effect size? I suspect Cohen's d?

**R2-9: Thank you for mentioning this. It should be indeed Cohen’s d. We added this information under Table 1 and Table 2.**
